# Cross-Sectional and Longitudinal Association between Neighborhood Environment and Perceived Control in Older Adults: Findings from HRS

**DOI:** 10.3390/ijerph182111344

**Published:** 2021-10-28

**Authors:** Sunwoo Lee

**Affiliations:** Faculty of Physical Education and Sport, Charles University, 162 52 Prague, Czech Republic; lee@ftvs.cuni.cz; Tel.: +420-777-316-761

**Keywords:** neighborhood characteristics, neighborhood, social cohesion, perceived control, older adults

## Abstract

The current study examined how neighborhood environments are related to older adults’ perceived control over time. A longitudinal study design was employed using data sampled from the Health and Retirement Study (HRS) 2014 and 2018. In total, 3170 older adults, whose age ranged between 60 and 99 years at the baseline, were followed up with a 4-year lag. Measures included two domains of neighborhood characteristics: social cohesion and physical disorder (at baseline and follow-up) and perceived control (at follow-up). Path coefficients between the latent factors were examined using structural equation modeling. Results showed that there was a significant cross-sectional and longitudinal association between neighborhood social cohesion and older adults’ perceived control, while neighborhood physical disorder was cross-sectionally associated with perceived control. Study findings provide evidence for promoting social integration and social capital in their neighborhood that might contribute to older adults’ perceived competence and beliefs in control.

## 1. Introduction

The existing literature in and beyond environmental gerontology has provided empirical evidence for the significant role of residential setting or neighborhood environments in older adults’ active and healthy aging [1,2,3,4,5,6]. In particular, (neighborhood) environmental correlates of older adults’ mental health outcomes and psychological well-being have been greatly examined [7,8,9,10,11,12,13]. These previous studies suggest several pathways of how neighborhood environments may determine older adults’ physical health and psychological well-being. Favorable neighborhood environments can promote older adults’ health behaviors and perception. For example, high walkability and accessibility to local parks may significantly facilitate participation in physical activity. Likewise, socially inclusive neighborhoods can promote older adults’ positive affect and subjective well-being and thereby enhance psychological resilience and personal coping strategies for managing aging-related declines and losses.

A handful of studies suggested that control beliefs might play a role in linking the neighborhood environments and health and quality of life outcomes. According to Ruiz et al. [11], perceived control significantly mediated the relationship between social cohesion in the neighborhood and depression in older adults from England, Czech, Poland, and Russia. Similarly, Zhang et al. [14] found that neighborhood characteristics, such as access to public space and senior services, facilitated older adults’ resilience, which, in turn, contributed to their emotional well-being, life satisfaction, and positive aging perception. However, relatively little studies have explicitly examined how neighborhood environments are associated with older adults’ perceived control over time, and thus the current study aims to address this gap in the literature.

Perceived control refers to a person’s beliefs about her own capability and available resources to control stressful life events or undesirable situations [15,16,17]. In a large body of literature, perceived control appeared to be associated with the psychological and physical health of older adults [18,19,20,21,22]. Some studies further revealed that control beliefs played a role in weakening the negative relationship between aging-related adversities and health and quality of life [23,24,25]. For example, Turiano et al. [26] found a significant moderating effect of perceived control on mortality risk among older adults with a low education level, while there was no significant association between control beliefs and mortality in older adults at a higher education level. This might suggest that perceived control has the potential to help lower socio-economic status (SES) older adults better maintain their health and pursue well-being. If neighborhood environments play an important role in explaining older adults’ control beliefs, we should better incorporate this understanding into implementing and evaluating a community intervention in the context of the neighborhood environment.

**Figure 1:**
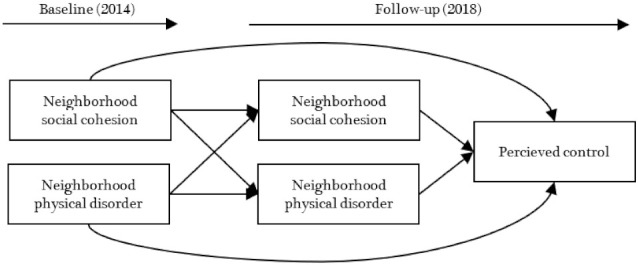
A hypothesized longitudinal model of neighborhood characteristics and perceived control.

The current study pointed to older adults’ perceived control and aimed at examining how neighborhood environments are related to older adults’ control beliefs over time. Figure 1 presents a study design including cross-sectional and longitudinal association between neighborhood social cohesion and physical disorder, and perceived control over a 4-year follow-up. It was first hypothesized that neighborhood social cohesion and physical disorder at the base year have significant auto-regressive and cross-lagged effects on neighborhood social cohesion and physical disorder at follow-up. It was also hypothesized that neighborhood social cohesion and physical disorder at the base and terminal year are significantly associated with perceived control at follow-up. Cross-lagged panel analysis of the current study will provide a better understanding of how different neighborhood characteristics affect each other over time, and further develop empirical evidence for potential causal influences between the neighborhood environments and perceived control. This will also add to existing literature by determining which neighborhood characteristic may be more likely to affect older adults’ perceived control.

## 2. Materials and Methods

### 2.1. Study Design and Sample Frame

A two-wave longitudinal study was employed using data sampled from the Health and Retirement Study (HRS), with data collected in 2014 (Time 1) and in 2018 (Time 2), which indicated a 4-year lag. Data is publicly available via https://hrsonline.isr.umich.edu/ (accessed on 10 December 2020) and a detailed description of the main survey design, data collection procedure, and obtained informed consent can be found elsewhere [27,28,29]. Due to the current analysis using a publicly available database, the Ethic Committee of the author’s institution waived the need for approval.

The study sample comprised 3170 United States older adults who completed leave-behind survey questionnaires in both data collections. The respondents’ age ranged between 60 and 99 years old (mean = 71.74, SD = 7.76) at the point of time 1 and between 65 and 104 years old (mean = 75.84, SD = 7.76) at the follow-up point. Final sample data comprised 59.1% of women. Caucasians accounted for 75.4%, 17.7% were Black or African Americans, and 6.9% were other (i.e., Native Americans, Asians); 12.7% considered themselves Hispanics. Of the respondents, 47.3% reported they attained a high school diploma, 18.5% maintained a two or four year college degree, 10.7% maintained master and professional degrees, 18.1% had no degree, 4.5% completed the General Educational Development (GED), and 0.9% reported degree unknown or some college (see Appendix A).

In relation to marital status and living arrangement, the structure of the sample slightly changed across times 1 and 2; this change in proportion was mostly due to bereavement. With respect to marital status, the following categories were used: being married (57.8% to 50.7%), separated/divorced (15.7% to 16.4%), widowed (23.0% to 28.5%), and never married (3.3% to 4.0%). In relation to living arrangement status, the following categories were used: living with partner (61.0% to 55.1%), married but not living with spouse/partner (2.5% to 0.6%), not married/partnered and living with other unrelated adults (1.1% to 1.2%), not married/partnered and living with relatives including minor children (11.2% to 13.3%), and living alone (24.0% to 29.5%).

### 2.2. Measurement

Two domains of neighborhood characteristics were assessed: social cohesion and physical disorder. Social cohesion refers to perceived social integration and trust within the neighborhood, whereas neighborhood physical disorder involves unfavorable physical indications of the neighborhood and safety of the neighborhood [30,31]. The study respondents were asked how they felt about their local area that was everywhere within a 20-min walk or about a mile of their home. Neighborhood social cohesion was measured using four questionnaire items (e.g., “feel part of this area”) using a 7-point Likert scale, where answers ranged between 1 “strongly disagree” and 7 “strongly agree”. A higher score indicated a greater level of social cohesion. Neighborhood physical disorder was measured using an additional four items (e.g., “no problem with vandalism and graffiti in this area”). Using a 7-point Likert scale, respondents selected an answer ranging from 1 “strongly disagree” to 7 “strongly agree”. A higher score indicated a lower level of disordered neighborhoods.

Perceived control was measured using a total of five multi-item scales [15,16]. Respondents were asked to indicate how much they agree or disagree with the given statements (e.g., “What happens in my life is often beyond my control”). Using a 6-point Likert scale, respondents selected an answer ranging from 1 “strongly disagree” to 6 “strongly agree”. Items were reverse coded, so a higher score indicated a greater level of perceived control. Detailed information about the measurement items can be seen in Appendix B.

### 2.3. Analysis

Descriptive and correlation analyses were performed to understand the structure of the sample data and association between latent factors. In order to examine study hypotheses, standardized estimates of significant path coefficients between the latent factors were examined using structural equation modeling (SEM). Goodness-of-fit-indices of the final structural model were reported. Fit indices included RMSEA (root mean square error of approximation), NNFI (non-normed fit index), CFI (comparative fit index), and AIC (Akaike information criterion) [32]. Demographic variables, such as gender, age (in born year), and education, were included to control for possible effects on the relationships between study variables. Gender was coded as 1 for men and 2 for women and education was coded as 0 for no degree, 1 for General Education Degree, 2 for high school diploma, 3 for two year college degree, 4 for four year college degree, 5 for master degree, and 6 for professional degrees. IBM SPSS Statistics 20.0 (IBM Corporation, Armonk, NY, USA)and Mplus version 8.4 software (Muthen & Muthen, Los Angeles, CA, USA)were used throughout the data preparation and analyses.

## 3. Results

### 3.1. Descriptive Statistics and Correlation Test

Table 1 provides descriptive statistics and composite reliability of the latent factors. Measures of the perceived neighborhood environment indicated an acceptable degree of internal consistency; Cronbach’s alpha values of 0.869 and 0.874 for social cohesion at times 1 and 2, respectively; and Cronbach’s alpha values of 0.841 and 0.848 for physical disorder at times 1 and 2, respectively. The construct of perceived control also indicated a good internal consistency and a Cronbach’s alpha value of 0.860.

Table 1 also provides information about the bivariate correlations between the latent factors. Five latent factors were weakly to strongly correlated with one other (0.143 to 0.777). The cross-sectional correlation between social cohesion and physical disorder was greatly significant, r = 0.777, at baseline and, r = 0.758, at follow-up. Longitudinal correlations were also significant across times 1 and 2. In addition, the variance inflation factor (VIF) was examined to determine if the sample data met the assumption of collinearity. Results indicated that VIF values were less than 10 (3.01 to 3.17), thus allowing for no problems related to multicollinearity in the analysis.

### 3.2. Structural Model Test

**Figure 2:**
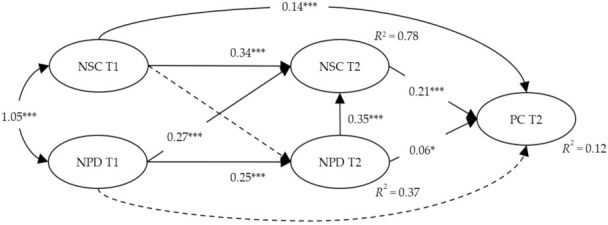
A final structural model with standardized estimates of significant path coefficients. *** *p* < 0.001, * *p* < 0.05. NPD: Neighborhood Physical Disorder; NSC: Neighborhood Social Cohesion; PC: Perceived Control. T1 = Time 1, T2 = Time 2. Two-headed connection indicates covariance between constructs. One-headed connectors indicate a causal path. The dotted line indicates that there was no significant path between the latent factors. Final structural model includes control variables (i.e., gender, age, and education) that are allowed to have effects on perceived control at follow-up.

A structural model with five latent factors was examined while controlling for the effects of gender, age, and education. Figure 2 visualizes the final structural model with significant path coefficients between the latent factors. Results indicated that there was a significant auto-regression and cross-lagged coefficient between the measures of neighborhood characteristics at times 1 and 2. Specifically, neighborhood social cohesion at baseline was significantly related to time 2 social cohesion (β = 0.34, SE = 0.02, *t*-value = 27.54, *p* < 0.001), and neighborhood physical disorder at baseline was significantly related to physical disorder at time 2 (β = 0.25, SE = 0.03, *t*-value = 16.54, *p* < 0.001). Although neighborhood physical disorder at baseline was significantly related to social cohesion at time 2 (β = 0.27, SE = 0.02, *t*-value = 18.53, *p* < 0.001), social cohesion at baseline did not have any cross-lagged effect on physical disorder at time 2.

Cross-sectional and longitudinal associations between neighborhood environments and perceived control varied. Neighborhood social cohesion at time 1 was positively related to perceived control at follow-up (β = 0.14, SE = 0.03, *t*-value = 4.54, *p* < 0.001), while physical disorder at time 1 was not related to perceived control at follow-up. Cross-sectional association between neighborhood social cohesion and physical disorder, and perceived control was all significant (β = 0.21, SE = 0.02, *t*-value = 8.54, *p* < 0.001, and β = 0.06, SE = 0.02, *t*-value = 2.60, *p* < 0.05, respectively). In addition, neighborhood physical disorder at time 2 significantly predicted social cohesion at time 2 (β = 0.35, SE = 0.02, *t*-value = 24.91, *p* < 0.001). The goodness-of-fit-indices of the final structural model indicated an acceptable fit to the sample data, χ^2^ = 974.699 (df =72, *p* < 0.001), CFI = 0.943, NFI = 0.920, RMSEA = 0.053.

## 4. Discussion

The current study examined how two aspects of neighborhood context are related to older adults’ perceived control over time. Results indicated that individual differences in perceived neighborhood social cohesion and physical disorder were relatively stable over the 4-year lag. However, different aspects of the neighborhood environment and its association with control beliefs varied. Results revealed a significant cross-sectional and longitudinal association between neighborhood social cohesion and perceived control. The effect of neighborhood social cohesion on perceived control remained significant after controlling for demographic variables. A handful of previous studies provided evidence for neighborhood social cohesion linked to psychological resources. Ruiz et al. [11], for example, showed that neighborhood social cohesion significantly predicted older adults’ perceived control of life and control at home. According to Kowitt et al. [8], neighborhood social cohesion, access to neighborhood resources, and safety were significantly related to perceived individual control over life, which, in turn, affected depression among adults aged 50 years and older.

Our results, together with previous findings, highlight the importance of social integration and social capital in the neighborhood. Cramm et al. [2] found that neighborhood social integration was positively associated with a greater level of physical and social well-being among older adults in the Netherlands. Similarly, Bromell and Cagney [33] suggested that neighborhood social cohesion allowed older adults, in particular, those who live alone, to more frequently experience companionship with others. Socially cohesive and favorable neighborhoods might facilitate mobility and social interaction among older adults, thus further promoting the perception that older adults have a social network and support that would help them cope with constraints.

In addition, neighborhood physical disorder was cross-sectionally associated with perceived control, but the effect was not considerable. The longitudinal association between physical disorder and perceived control was not statistically significant. According to Lee [4], for example, neighborhood social cohesion was significantly related to walking behavior and positive affect in older adults, but the effect of physical disorder tended to be alleviated due to the effect of social cohesion. However, this does not necessarily de-emphasize the role of neighborhood physical disorder in promoting older adults’ psychological resources. According to the results, neighborhood physical disorder was significantly related to social cohesion over time. More specifically, neighborhood physical disorder at baseline significantly predicted neighborhood social cohesion at follow-up, while neighborhood social cohesion at baseline did not predict physical disorder at follow-up. Furthermore, cross-sectional observation at the follow-up point also indicated that neighborhood physical disorder influenced social cohesion, not vice versa. Specifically, physical disorder indications should be equally or even preferentially managed to increase neighborhood social cohesion that spills over and has a positive effect on older adults’ psychological agency.

## 5. Conclusions

Study findings reinforced the importance of neighborhood contexts that are relevant to older adults’ psychological competence and beliefs in control. Results also stressed social integration and social capital in the neighborhood by providing empirical evidence for cross-sectional and longitudinal association between social cohesion and perceived control. Based on study findings, future studies should examine if neighborhood social cohesion mediates the relationship between neighborhood physical disorder and perceived control. This will help better contextualize the different roles of neighborhood characteristics in promoting older adults’ personal and psychological resources. Furthermore, future studies should reiterate our final structural model by controlling additional correlates, such as health status and functional limitations, as these variables are likely to affect older adults’ sense of control and level of dependence on the resources out of their home environment.

## Figures and Tables

**Table 1:** Descriptive statistics and correlations.

	Mean (SD)	Cronbach’s *a*	1	2	3	4	5
1.NPD (T1)	2.52 (1.43)	0.841	-				
2.NSC (T1)	2.59 (1.40)	0.869	0.777 **	-			
3.NPD (T2)	2.40 (1.38)	0.848	0.404 **	0.324 **	-		
4.NSC (T2)	2.52 (1.35)	0.874	0.298 **	0.403 **	0.758 **	-	
5.PC (T2)	4.74 (1.12)	0.860	0.143 **	0.204 **	0.186 **	0.239 **	-

Note: ** *p* < 0.01. NPD: Neighborhood Physical Disorder; NSC: Neighborhood Social Cohesion; PC: Perceived Control. T1 = Time 1, T2 = Time 2.

## Data Availability

HRS data are publicly available via https://hrsonline.isr.umich.edu/ (accessed on 10 December 2020).

## Appendix A

**Table A1:** Baseline sample characteristics (*n* = 3170).

		%
Gender	Female	59.1
Age		Mean = 71.74 years, SD = 7.76
Education	No degree	18.1
	General Educational Development (GED)	4.5
	High school diploma	47.3
	Two-year college degree	4.9
	Four-year college degree	13.6
	Master’s degree	8.1
	Professional degree	2.6
Race/ethnicity	White/Caucasian	75.4
	Black/African American	17.7
	Other (American Indian, Alaskan Native, Asian, Native Hawaiian, and Pacific Islander)	6.9
Hispanicity type	Hispanic, Mexican	7.6
	Hispanic, other	4.9
	Hispanic, type unknown	0.2
	Non-Hispanic	87.2
Marital status	Married	57.9
	Separated/divorced	15.7
	Widowed	23.0
	Never married	3.3
Living arrangement	Married/partnered,	
	living with partner	61.0
	not living with spouse/partner	2.5
	Not married/partnered,	
	living with another unrelated adult	1.1
	living with relative (e.g., children)	11.2
	living alone	24.0

## Appendix B

**Table A2:** Items used to measure neighborhood social cohesion, neighborhood physical disorder, and perceived control.

Construct	Items
neighborhood environment—Physical disorder	There are no vacant or deserted houses or storefronts in this area.
	There is no problem with vandalism and graffiti in this area.
	This area is kept very clean.
	People feel safe walking alone in this area after dark.
neighborhood environment—Social cohesion	I really feel part of this area.
	Most people in this are friendly.
	Most people in this area can be trusted.
	If you were in trouble, there are lots of people in this area who would help you.
Perceived control	I often feel helpless in dealing with the problems of life.
	Other people determine most of what I can and cannot do.
	What happens in my life is often beyond my control.
	I have little control over the things that happen to me.
	There is really no way I can solve the problems I have.

Perceived neighborhood environment was measured using a 7-point Likert-type scale, from 1 (strongly disagree) to 7 (strongly agree). Perceived control was measured using a 6-point Likert-type scale, from 1 (strongly disagree) to 6 (strongly agree).
